# Horner syndrome as a postoperative complication after minimally invasive video-assisted thyroidectomy: A case report

**DOI:** 10.1097/MD.0000000000008888

**Published:** 2017-12-01

**Authors:** Xiaolei Hu, Xiaomei Zhang, Huaiyong Gan, Dajun Yu, Weihua Sun, Zhaoming Shi

**Affiliations:** a Department of Endocrinology; b Department of Pathology; c Department of Thyroid and Breast Surgery, The First Affiliated Hospital of Bengbu Medical College, Bengbu 233004, Anhui, China.

**Keywords:** Graves disease, Horner syndrome, minimally invasive thyroidectomy, papillary thyroid carcinoma, postoperative complication

## Abstract

**Rationale:**

Horner syndrome is an unusual complication after thyroidectomy.

**Patient concerns:**

We report a case of Horner syndrome in a 34-year-old female patient with Graves disease associated with papillary thyroid carcinoma who underwent left-side minimally invasive video-assisted thyroidectomy and neck dissection.

**Diagnosis:**

Horner syndrome was diagnosed based on left myosis, eyelid ptosis, and mild enophthalmos, which developed in the patient on postoperative day 2.

**Interventions:**

The patient was administered glucocorticoids and neurotrophic drugs on postoperative day 3.

**Outcome:**

The symptoms of Horner syndrome were significantly relieved 1 year later.

**Lessons:**

Surgeons must be aware that Horner syndrome may be a source of iatrogenic complications, and patients also should be informed of these complications before surgery.

## 1 Introduction

Horner syndrome (HS) is associated with a constellation of symptoms, including ipsilateral myosis, ptosis, enophthalmos, and facial anhidrosis, and is due to a disruption of any part of the ipsilateral sympathetic innervation.^[1,2]^ HS can be divided into 3 types according to the localization of the sympathetic lesion: central (first-order neuron), preganglionic (second-order neuron), and postganglionic (third-order neuron). The causes of HS include stroke in the posteroinferior cerebellar artery, trauma, tumors, cluster migraine, and carotid dissection.^[2,3]^ Although it is an unusual complication of thyroidectomy due to improvements in surgical techniques, HS is inevitable because of the close and highly variable anatomical relationship between the thyroid gland and cervical sympathetic trunk.^[4–7]^ Here, we report a case of HS in a patient with Graves disease associated with papillary thyroid carcinoma who underwent left-side thyroidectomy and neck dissection.

## 2 Case report

A 34-year-old female suffered from palpitations, weight loss, weakness, and fatigability for >1 month. Upon examination, she had a blood pressure of 103/58 mm Hg, pulse rate of 95 min^−1^, respiratory rate of 18 min^−1^, and grade-I thyroid enlargement with small thyroid nodules on the left side. She had no exophthalmos or any other positive signs. Laboratory findings showed a serum thyroid-stimulating hormone (TSH) level of 0.01 mIU/L (normal range: 0.4–4.0), total T4 level of 162 ng/mL (normal range: 45–125), total T3 level of 2.32 ng/mL (normal range: 0.58–1.69), and TSH receptor antibody (TRAb) level of 2.09 U/L (normal range: <1). Thyroid ultrasonography revealed that the left thyroid had a 7 × 8 × 8 mm nodule that was hypoechoic and irregular. Her Thyroid Imagine Reporting and Data System (TI-RADS) score was 4c. Based on the presence of thyrotoxicosis, an enlarged thyroid, high T4 and T3 levels, a low TSH level, and TRAb positivity, the patient was diagnosed with Graves disease. Because her thyroid nodules were classified as 4c, she was transferred to the Department of Thyroid and Breast Surgery for further treatment.

**Figure 1:**
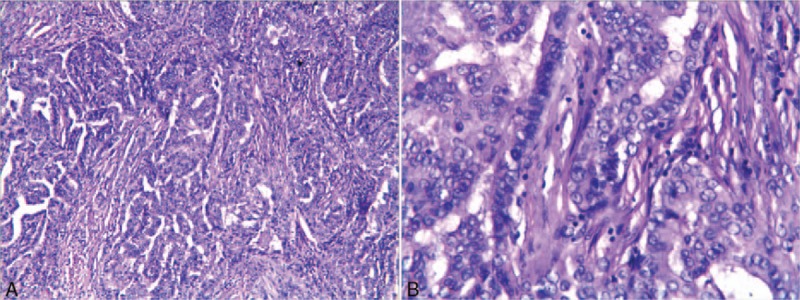
Left papillary thyroid microcarcinoma (HE staining): (A) ×100; (B) ×400.

**Figure 2:**
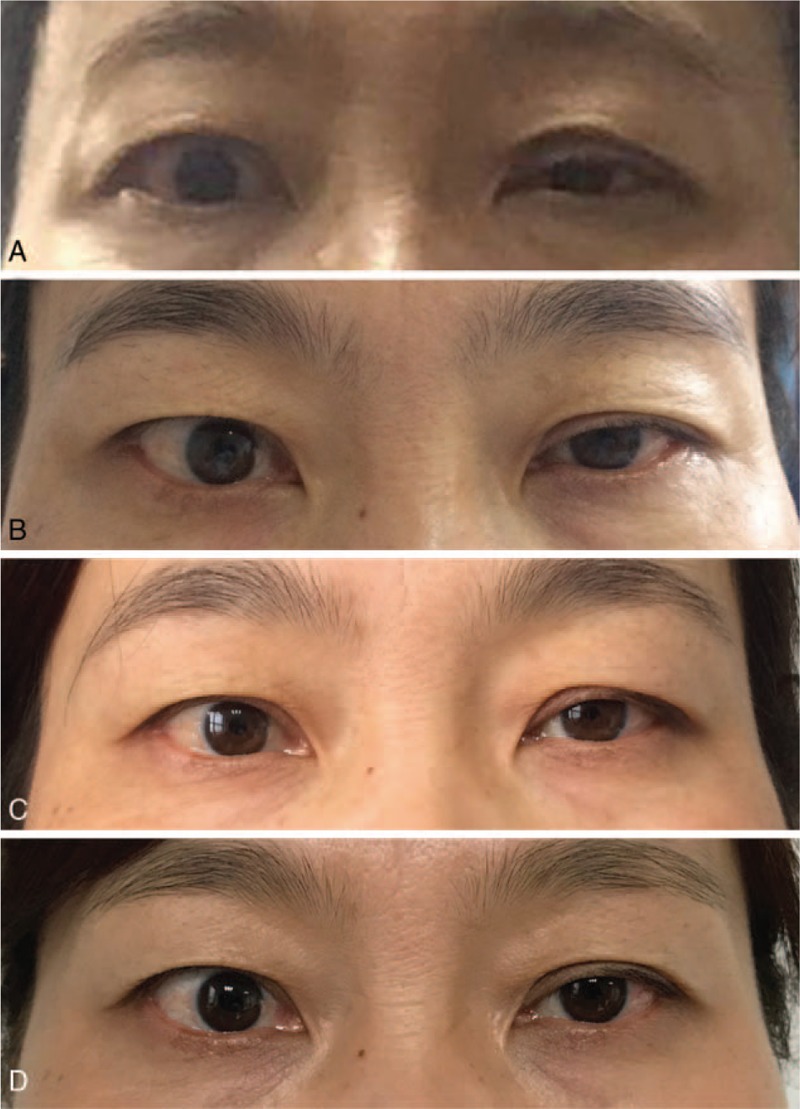
Patient with Horner syndrome. (A) Two days postoperative. (B) One month postoperative. (C) Six months postoperative. (D) One year postoperative.

The patient underwent left-side minimally invasive video-assisted thyroidectomy (MIVAT), prophylactic central and level I-V neck dissection through a small incision in the neck using an ultrasonic scalpel. A postoperative pathological examination revealed left papillary thyroid microcarcinoma (Fig. 1) and lymph node negativity for all nodes. On postoperative day 2, left myosis, eyelid ptosis, and mild enophthalmos developed (Fig. 2), without anhidrosis or vascular dilatation of the face. No other complications, such as bleeding, wound infection, vocal cord palsy, or hypoparathyroidism developed. On postoperative day 3, the patient was administered glucocorticoids (intravenous infusion of 10 mg dexamethasone once a day for 3 days) and neurotrophic drugs (intravenous infusion of 0.5 mg mecobalamin once a day for 7 days). After 1 year of follow-up, she showed a significant improvement in ptosis and myosis (Fig. 2), her thyrotoxicosis disappeared, and laboratory examinations were normal, except for inhibition of TSH (0.1 mIU/L, treated with 75 μg levothyroxine sodium).

## 3 Discussion

MIVAT was first used in 1998.^[8]^ Subsequently, this surgical procedure has become widely performed due to its satisfactory cosmetic result and the minor pain experienced by the patients.^[9]^ Although the surgical techniques have improved continuously, sporadic cases of HS development as a postoperative complication of MIVAT have been reported.^[6,10]^ HS following thyroidectomy is a very rare complication. A retrospective study conducted in Korea found that the incidence rate of HS was 0.2% in all patients,^[11]^ if the operative methods were not considered. Another retrospective study with 3000 patients evaluated surgical complications after robotic thyroidectomy for thyroid carcinoma and showed that the incidence rate of HS was 0.03%.^[12]^ In addition, Chinese populations had a lower incidence rate based on a recent study, which found that among the 416 patients who received MIVAT, HS was postoperatively identified in 2 female patients.^[6]^

There are several possible mechanisms of HS after thyroidectomy. Mechanical injury during surgery: Our patient underwent MIVAT, which is minimally invasive, but the small incision on the neck might need to be retracted more forcefully to laterally expose the carotid sheath. This procedure may result in hematoma, ischemia, and edema-induced transient neural damage.^[13,14]^ Injury of the middle cervical ganglion: The middle cervical ganglion and sympathetic trunk are in close proximity and have a variable relationship, located either in front or behind the inferior thyroid artery as the artery arches medially from the thyrocervical trunk. This close and, more importantly, variable anatomical relationship makes the sympathetic trunk and the middle cervical ganglion highly susceptible during thyroidectomy.^[7]^ In addition, the inferior thyroid artery has been reported to supply the sympathetic chain, which can be affected due to ligation of this artery.^[6]^ Heat injury induced by the ultrasonic scalpel: In our report, the operation was performed with an ultrasonic scalpel. During the dissection of the lymphatic adipose tissue on the medial border of the common carotid artery, the cervical sympathetic trunk was injured by the heat conduction because the ultrasonic scalpel was near the cervical sheath.

To the best of our knowledge, this is the first case of HS reported in a patient with Graves disease associated with papillary thyroid carcinoma after MIVAT in which treatment and follow-up were conducted for 1 year. In our report, the patient was administered glucocorticoids (dexamethasone) and neurotrophic drugs (mecobalamin), and she showed a significant improvement after 1 year. Therefore, we speculate that these drugs may reduce edema-induced neural damage and promote neural repair.

Although HS is rare, surgeons must be aware that it may be a source of iatrogenic complications, and patients should also be informed of these complications before surgery. When the surgeons perform neck dissection, especially central neck dissection, they should protect the cervical sympathetic nerve. If damage occurs, neurotrophic drugs and glucocorticoids may be an appropriate treatment.

## Acknowledgment

The authors would like to thank the patient featured in this case report.
